# The mechanism of HMGB1 secretion and release

**DOI:** 10.1038/s12276-022-00736-w

**Published:** 2022-02-25

**Authors:** Ruochan Chen, Rui Kang, Daolin Tang

**Affiliations:** 1 Department of Infectious Diseases, Xiangya Hospital, Central South University, Changsha, Hunan 410008 China; 2 Hunan Key Laboratory of Viral Hepatitis, Xiangya Hospital, Central South University, Changsha, Hunan 410008 China; 3 Department of Surgery, UT Southwestern Medical Center, Dallas, TX USA

**Keywords:** Molecular biology, Medical research

## Abstract

High mobility group box 1 (HMGB1) is a nonhistone nuclear protein that has multiple functions according to its subcellular location. In the nucleus, HMGB1 is a DNA chaperone that maintains the structure and function of chromosomes. In the cytoplasm, HMGB1 can promote autophagy by binding to BECN1 protein. After its active secretion or passive release, extracellular HMGB1 usually acts as a damage-associated molecular pattern (DAMP) molecule, regulating inflammation and immune responses through different receptors or direct uptake. The secretion and release of HMGB1 is fine-tuned by a variety of factors, including its posttranslational modification (e.g., acetylation, ADP-ribosylation, phosphorylation, and methylation) and the molecular machinery of cell death (e.g., apoptosis, pyroptosis, necroptosis, alkaliptosis, and ferroptosis). In this minireview, we introduce the basic structure and function of HMGB1 and focus on the regulatory mechanism of HMGB1 secretion and release. Understanding these topics may help us develop new HMGB1-targeted drugs for various conditions, especially inflammatory diseases and tissue damage.

## Inflammation: Nuclear protein offers target for controlling immune response

A nuclear protein that gets released after cell death or is actively secreted by immune cells offers a promising therapeutic target for treating diseases linked to excessive inflammation. Daolin Tang from the University of Texas Southwestern Medical Center in Dallas, USA, and colleagues review how cellular stresses can trigger the accumulation of HMGB1, a type of alarm signal protein that promotes the recruitment and activation of inflammation-promoting immune cells. The researchers discuss various mechanisms that drive both passive and active release of HMGB1 into the space around cells. These processes, which include enzymatic modifications of the HMGB1 protein, cell–cell interactions and molecular pathways of cell death, could be targeted by drugs to lessen tissue damage and inflammatory disease caused by HMGB1-induced immune responses

## Introduction

In 1973, Ernest Johns, Graham Goodwin and colleagues extracted a set of nonhistone proteins from calf thymus chromatin^1,2^. Subsequently, these proteins were named “high mobility group” (HMG) proteins because of their high mobility in polyacrylamide gel electrophoresis systems with no signs of aggregation. Currently, HMG family proteins include HMGB, HMGN, and HMGA subfamilies, which are highly evolutionarily conserved nuclear proteins^3^. As the most abundant protein among all the HMG family members and the second-most abundant protein in the nucleus, HMGB1 (also known as HMG1^4^, HMG-1^5^, and amphoterin^6^) is widely expressed in mammalian cells and tissues^7^. Normally, HMGB1 is mainly located in the nucleus and binds to chromatin, but it can shuttle from the nucleus to the cytoplasm under various stress conditions and then into the extracellular space, for example, when induced by elevated reactive oxygen species (ROS) production during porcine circovirus type 2 infection^8,9^. The subcellular location of HMGB1 varies depending on cell type, tissue, and stress signals, and its location is key to its functions^10,11^. HMG proteins have multiple nuclear functions, and additional extracellular and proinflammatory activities are continuously being discovered for HMGB1 ^5,12^and related proteins, such as HMGB2^13^ and HMGN1^14^. In response to infection and tissue damage, HMGB1 can be actively secreted and passively released outside cells, where they function as damage-associated molecular pattern molecules (DAMPs) to mediate inflammation and immune responses^15,16^. In this minireview, we briefly introduce basic HMGB1 biology and focus on the mechanisms involved in HMGB1 secretion and release in the context of stressors. Gaining knowledge of HMGB1 may help us develop new HMGB1-targeted drugs for disease treatment.

## HMGB1 structure

**Fig. 1:**
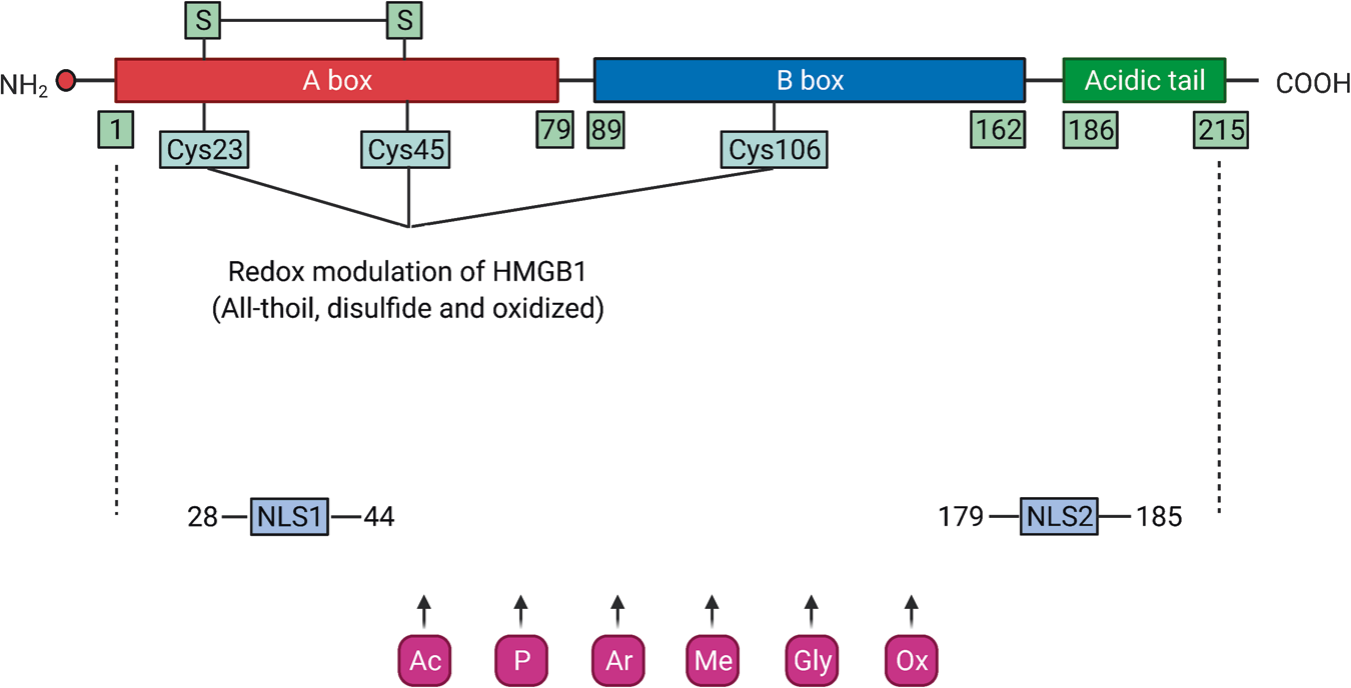
The structure and modification of HMGB1. HMGB1 consists of three domains: two DNA binding domains (Box A and Box B), a C-terminus and an N-terminus. HMGB1 undergoes various posttranslational modifications after cell activation induced by external stimuli, resulting in its translocation from the nucleus to the cytoplasm and finally its release. Ac, acetylation; Ar, ADP-ribosylation; Cys, cysteine; Gly, N-glycosylation; Me, methylation; NLS1/2, nuclear localization signal 1/2; Ox, oxidation; P, phosphorylation.

The mRNA of HMGB1 is polyadenylated^17^, and the protein sequence of HMGB1 shows 100% homology between mice and rats and 99% homology between rodents and humans^18^. The human HMGB1 protein consists of 215 amino acid residues, which are arranged in two consecutive DNA binding domains (namely, the HMG A box domain [9–79 amino acids] and HMG B box domain [95–163 amino acids]) followed by a C-terminal acidic tail (186–215 amino acids) and a short but functionally significant N-terminal region. The two HMG boxes of HMGB1 are structurally composed of three alpha helices and two loops that are arranged in an “L” shape^19^. The steady location of HMGB1 in the nucleus is due to two nuclear localization signals (NLSs): NLS1 (28–44 amino acids) and NLS2 (179–185 amino acids)^20^, while the nuclear export signal (NES) is contained in the DNA binding domains. Thus, an abnormal HMGB1 subcellular location can be induced with changes in the NES and NLS^2^. Specific residues in the HMGB1 sequence are responsible for the interaction, binding, activity, and function of HMGB1^19,21,22^. The extracellular B box can recapitulate proinflammatory activity, whereas the A box can act as an HMGB1 antagonist due to its ability to bind to the B box^23^. After fusion with the C-terminal acid tail, the anti-inflammatory activity of the HMGB1 A box is enhanced^24^. Overall, the structure of HMGB1 is highly conserved across species (Fig. 1).

## HMGB1 modification

The position and activity of HMGB1 are affected by posttranslational modifications. HMGB1 can shuttle between the nucleus and the cytoplasm by modifying NLS1 and NLS2 through acetylation and deacetylation mediated by histone acetyltransferase (HAT) family proteins and histone deacetylase (HDAC) family proteins^12,20,25^. In contrast, acetylation of lysine in the NLS prevents nuclear reentry of HMGB1^20,26^. In addition, HMGB1 can bind with exportin 1 (XPO1, best known as chromosome region maintenance 1 [CRM1]) and be acetylated under oxidative stress and then transferred to the cytoplasm^27^. Animal studies have confirmed that various oxidative stresses can induce the release of hyperacetylated HMGB1 under various pathological conditions^28,29^. Various signaling pathways are implicated in HMGB1 acetylation and deacetylation, such as the interferon (IFN)-Janus kinase (JAK) signal transducer and activator of transcription 1 (STAT1) pathway^30,31^ and sirtuin1 (SIRT1) pathway^32^. In addition to acetylation^33^, other modifications, such as methylation, N-glycosylation, phosphorylation, and oxidation, can regulate the translocation and release of HMGB1 to the extracellular space in response to various stresses^20,27,34–40^. For example, phosphorylation of the serine residues of NLS1 and NLS2, which is mediated by protein kinase C (PKC), regulates the nucleus-cytoplasm shuttling of HMGB1^38^. Lys42 methylation of nuclear HMGB1 can alter HMGB1 protein conformation and reduce HMGB1–DNA binding activity^35,41^, leading to passive diffusion of HMGB1 out of the nucleus. Secretion of HMGB1 from macrophages can be facilitated when HMGB1 is poly(ADP-ribosyl)ated, a posttranslational modification produced by poly(ADP-ribose) polymerase 1 (PARP1)^37,42^. Glutamate residues at 40, 47, and 179 have been shown to be possible ADP-ribosylation sites^43–45^. Liquid chromatography tandem mass spectrometry showed that N-glycosylation of three residues of HMGB1 (N37, N134, and N135) attenuates the binding of HMGB1 with DNA and promotes HMGB1 release^39^. HMGB1 possesses three cysteine residues at amino acids C23, C45, and C106. Three redox forms of HMGB1 depend on the redox conditions of the environment and are associated with its function^26,46^. Intracellular fully reduced HMGB1 (fr-HMGB1), with three conserved cysteines containing thiol groups, can form a complex with CXCL12 and promote the migration of immune cells. Partially reduced HMGB1 with a disulfide bond between cysteine 23 and cysteine 45, termed disulfide HMGB1 (ds-HMGB1), triggers inflammatory responses. Fully oxidized HMGB1 (ox-HMGB1) has no chemokine or cytokine activity^26,46^. According to the redox status of these residues, HMGB1 can induce or suppress the immune response through its subcellular location^40^. In summary, various modifications mediate the translocation of HMGB1 from the nucleus to the cytoplasm and then to the extracellular space (Fig. 1 and Table 1). However, whether and how these posttranscriptional modifications are competitively, cooperatively, or independently regulated under different conditions remains obscure^7^.

**Table 1:** Sites and functions of posttranslational modification (PTM) of HMGB1.

PTM	Site	Function
Acetylation/deacetylation	Lysine in NLS	HMGB1 translocation between the nucleus and cytoplasm^12,20,25,34^
Methylation	Lys42	Alters HMGB1 protein conformation and reduces its DNA binding activity^35,41^
Phosphorylation	Serine residues in NLS1 and NLS2	Regulates the nucleus-cytoplasm shuttling of HMGB1^36,38^
ADP-ribosylation	C-terminal tail	Promotes HMGB1 secretion in macrophages^37,42–45^
N-glycosylation	N37, N134, and N135	Attenuates the binding of HMGB1 with DNA and promotes HMGB1 release^39^
Redox and oxidation	C23, C45, and C106	Regulates the inflammatory activity of HMGB1^40^; induces translocation of HMGB1 protein from the nucleus to the cytoplasm^53–61^

## Active HMGB1 secretion

**Fig. 2:**
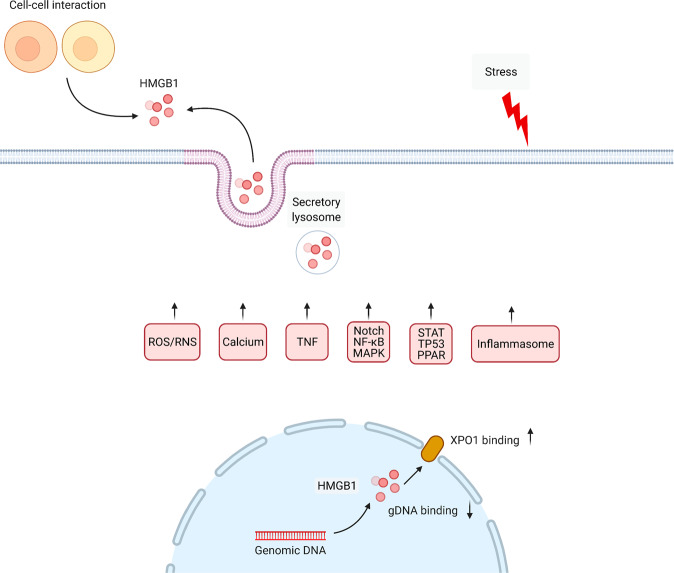
Active secretion of HMGB1 during stress. HMGB1 secretion is mediated by secretory lysosomes, which can be triggered by a variety of cytokines, signaling pathways, and cell–cell interactions.

Wang and colleagues first reported in 1999 that cultured macrophages treated with the endotoxin lipopolysaccharide (LPS) can secrete HMGB1 in large quantities into the extracellular space^5^. The release of HMGB1 was further confirmed in a mouse model of lethal endotoxemia^5^. In addition to LPS, accumulating evidence has documented that exogenous microbial products, various pathogen infections, and endogenous host stimuli can induce active HMGB1 secretion in multiple cells, such as, but not limited to, immune cells, fibroblasts, and epithelial or endothelial cells^47^. Due to the lack of a leader sequence, HMGB1 cannot be actively secreted through the conventional endoplasmic reticulum-Golgi secretory pathway that most soluble secretory proteins utilize^19,48^. Nevertheless, two models of HMGB1 active release have been proposed and frequently cited^49^. One involves activation of target cells with stimuli, resulting in secretion of HMGB1 into the extracellular space^20,50^. The second is packaging of HMGB1 into intracellular vesicles (such as lysosomes or autophagosomes) and subsequent release of HMGB1 outside the cell after fusion of the vesicles with the cell plasma membrane^25,51^. The detailed signals and modulators (proteins or organelles) involved in HMGB1 active secretion are described below (Fig. 2).

### ROS

ROS are oxygen atom-containing free radicals generated during various physiological processes and pathological disorders. The ROS family includes singlet oxygen (^1^O_2_^•^), superoxide anion (O_2_^•−^), hydrogen peroxide (H_2_O_2_), and hydroxyl radical (•OH). ROS generation is one of the basic mechanisms that causes the antibacterial activity of immune cells, and it has multiple roles in signal transduction. However, ROS are a main contributor to oxidative stress, and excessive ROS accumulation can lead to cell damage and cell death^52^. Mounting evidence has demonstrated the important role of ROS in the active secretion and passive release of HMGB1 in immune and nonimmune cells^53^. For example, paclitaxel increases ROS accumulation and phosphorylation of p38 mitogen-activated protein kinase 1 (MAPK1)-nuclear factor kappa B (NF-κB) signaling molecules, which leads to HMGB1 secretion from macrophages^54^. Cultured normal human melanocytes can release HMGB1 following treatment with H_2_O_2_^27,28,55^. H_2_O_2_ induces HMGB1 release in macrophages and monocytes through activation of the MAPK and NF-κB pathways^27,41^. Antioxidants, such as N-acetylcysteine^56^, quercetin^57^, ethyl pyruvate^54^, edaravone^58^, pyrrolidine dithiocarbamate^59^, EUK-8^60^, tert-butylhydroqinone^60^, and resveratrol^61^, significantly inhibit HMGB1 release in various infection and injury models in vitro and in vivo.

The key regulator of the response to oxidative stress is NFE2 like BZIP transcription factor 2 (NFE2L2, best known as nuclear factor erythroid 2-related factor [NRF2]), which acts as a transcription factor to trigger the expression of a series of antioxidant genes, such as heme oxygenase-1 (HMOX1, best known as HO-1)^62^. In addition to its primary function in heme homeostasis, HMOX1 also plays a vital role in antioxidant protection and the anti-inflammatory process of macrophages^63^. HMOX1 catalyzes the first and rate-limiting step in the oxidative degradation of heme to carbon monoxide, biliverdin, and ferrous iron. Biliverdin and carbon monoxide have anti-inflammatory properties. A dysfunctional NFE2L2-HMOX1-HMGB1 signaling pathway has been implicated in various pathological conditions, especially in hypoxia-induced injury in the heart, intestine, and brain^62,64–69^. Upregulation of HMOX1 inhibits the translocation of HMGB1 to the cytosol, thereby reducing its active secretion^64^. Hemin blocks radiation-induced HMGB1 release in an HMOX1-dependent manner in human keratinocyte HaCaT cells^70^. As expected, the absence of NFE2L2 reduces the expression of HMOX1 during inflammation and increases the release of HMGB1^70,71^. However, downregulation of HMGB1 is necessary for NFE2L2-mediated HMOX1 production during pulmonary fibrosis^72^. The activation of the NFE2L2-HMOX1-HMGB1 axis is further regulated by β-1-adrenergic receptors, leading to a reduction in cardiomyocyte damage caused by hypoxia/reoxygenation^68^. Overall, the mutual regulation between HMOX1 and HMGB1 signals is important for elucidating the feedback mechanism of oxidative stress. Further exploration of the collaboration between HMGB1 and NFE2L2-targeted genes in infection and innate immunity is important.

### RNS

Reactive nitrogen species (RNS), including nitric oxide (NO) and peroxynitrite (ONOO^-^), are important regulators of inflammatory diseases^73^. NO is a powerful vasodilator produced by NO synthase from L-arginine, O_2_, and nicotinamide adenine dinucleotide phosphate (NADPH). Although NO has an extremely short half-life in the blood, it plays a dual role in regulating cellular signal transduction during physiological and pathological processes. The production of NO is mediated by NO synthases, such as nitric oxide synthase 2 (NOS2, also known as iNOS), in response to endotoxins or cytokines, which contributes to killing or inhibiting the growth of invading microorganisms or neoplastic tissue. Carbon monoxide-releasing molecule 2 (CORM-2) specifically inhibits NOS2 expression and NO production, which blocks LPS-induced HMGB1 release in macrophages through the IFNB-JAK2-STAT1 pathway. In contrast, the presence of an NO donor (NOC-18) reverses this process, indicating a role of NO metabolism in regulating HMGB1 release^74^. As a feedback mechanism, HMGB1 inhibits NOS2 mRNA in a dose-dependent manner in the presence of IFNG and TNF, thereby antagonizing the immunosuppressive ability and therapeutic effects of mesenchymal stem cells during acute kidney injury^75^. HMGB1 also binds to toll like receptor 4 (TLR4) to inhibit nitric oxide synthase 3 (NOS3, also known as eNOS) expression and subsequent NO release in the setting of diabetes^76,77^. These findings highlight the complex relationship between HMGB1 and NO under various stresses.

During ischemia/reperfusion injury, explosive production of ONOO^-^ is rapidly induced by a surge in NO and O_2_^−^^78–80^. ONOO^−^ exhibits more potent toxicity than its precursor^81^. ONOO^−^ also mediates HMGB1 release and subsequent signaling pathways in different experimental models^82,83^. Free radicals or ONOO^−^ scavengers, such as edaravone, baicalin, and phenylboronic acid, effectively inhibit the translocation and release of HMGB1 as well as HMGB1-mediated production of proinflammatory cytokines^84–88^. In addition, HMGB1 promotes ONOO^−^ generation by activating NADPH oxidase via interaction with TLR4 or advanced glycosylation end-product specific receptor (AGER, best known as RAGE)^87,89–91^. However, there is still a lack of clear evidence to clarify the interaction between HMGB1 and RNS signaling pathways, which requires further in-depth investigations.

### Calcium ions

As a universal second messenger, calcium ions are involved in a wide range of cellular processes by exerting allosteric regulatory effects on intracellular enzymes or proteins^92^. The release of calcium ions and their transport between the cytoplasm and intracellular storage are regulated by numerous proteins, channels, and pumps^93–95^. Disrupted intracellular calcium signaling leads to severe cell damage and even cell death^96–98^. Calcium-mediated signal transduction is implicated in HMGB1 translocation and release during infection^99^, sterile inflammation^100,101^, and cancer^102,103^. Of note, the phosphorylation and release of HMGB1 are regulated by the activation of calcium-mediated protein kinases, especially Ca^2+^/calmodulin-dependent protein kinase kinase (CaMKK)^92,102,104,105^. In addition, IFNB regulated by PKC activity and calcium signaling pathways is also involved in the release of HMGB1^106^. Consequently, calcium signaling inhibitors (e.g., STO609, CV159, 2-APB, and U73122), calcium chelators (e.g., BAPTA) or knockdown/knockout of CaMKK inhibit HMGB1 secretion and protect animals in various disease models^99,107^. Further determining whether different sources of calcium play a similar role in mediating HMGB1 secretion in activated immune cells is necessary.

### XPO1

XPO1 is a member of the importin β superfamily of nuclear transport receptors involved in centrosome duplication and spindle assembly during mitosis^108^. XPO1 recognizes and exports proteins containing a leucine-rich NES, acting as a nuclear transport receptor engaged in extranuclear transportation of proteins or RNA^108,109^. Redistribution of HMGB1 is also strictly regulated by XPO1 in response to various inflammatory stimuli^40^. XPO1 inhibitors significantly inhibit HMGB1 translocation from the nucleus to the cytoplasm^110,111^. In contrast, the molecular chaperone heat shock protein family A (Hsp70) member 1A (HSPA1A, also known as HSP72) inhibits the interaction between HMGB1 and XPO1, thereby blocking HMGB1 secretion in macrophages following treatment with LPS or tumor necrosis factor (TNF)^112^. In addition, N-linked glycosylation of HMGB1 is crucial for the HMGB1-XPO1 interaction and nucleocytoplasmic transport as well as for extracellular secretion during inflammation^39^. XPO1-mediated nuclear export of tumor suppressor proteins has been implicated in tumorigenesis and drug resistance in several cancer types^113,114^. Since the release of HMGB1 plays an important role in shaping the tumor immune microenvironment, it is also expected that XPO1 can promote the translocation and release of HMGB1 in cancer.

### TNF

Macrophages and monocytes release TNF and HMGB1 in a time-dependent manner in response to LPS and interferon gamma (IFNG, also known as IFN-γ) stimulation^5,115,116^. Direct TNF suppression via genetic TNF knockout or TNF-neutralizing antibodies partially inhibits IFNG- and LPS-induced HMGB1 release in macrophages^116^, suggesting that HMGB1 secretion is partially mediated through a TNF-dependent mechanism. In addition, CD14 and Janus kinase 2 (JAK2) act as upstream regulators in a TNF-mediated HMGB1 release signaling pathway in response to LPS and IFNG, respectively^116^, highlighting the notion that an integrated signaling pathway controls HMGB1 secretion in activated macrophages.

### NF-κB

As a classical inflammatory signaling pathway, NF-κB plays a crucial role in the complex regulatory network of inflammation and immunity through the production and secretion of multiple proinflammatory cytokines and chemokines^117^. In the classical NF-κB activation pathway, subunit p65/p50 dimers are activated in response to various stress signals (such as cytokines, LPS, growth factors and antigen receptors). After inhibitor of NF-κB beta (IKBKB) protein is phosphorylated, its subsequent degradation by the proteasome leads to translocation of NF-κB p65/p50 to the nucleus, thereby inducing target gene expression alone or in combination with other transcription factor families. The NF-κB pathway is involved in HMGB1 release, whereas inhibition of the canonical NF-κB pathway limits HMGB1 secretion in activated immune cells^118–120^, although the NF-κB target gene directly responsible for this process is still u nclear. One possibility is that, as mentioned above, TNF (the classic NF-κB target gene) may be involved in the NF-κB-dependent release of HMGB1.

### Notch

As an evolutionarily conserved pathway, the Notch signaling cascade plays a vital role in various developmental and physiological processes, regulating cell fate, proliferation, survival, and homeostasis^121^. There are four Notch receptors (Notch 1 to 4) and five Notch ligands (Delta-like 1, 3, and 4 and Jagged canonical Notch ligand 1/2 [Jagged 1 and 2]) in mammalian cells. Dysregulation of the Notch-mediated signaling pathway is also correlated with human diseases and pathological conditions^121^. For example, impaired Notch signaling is involved in inflammatory disorders and inflammatory cytokine release^122–125^. Notch signaling is activated after LPS stimulation, and Jagged 1 expression is increased in a JNK-dependent manner. In addition, in vitro and in vivo, Notch signaling can be inhibited by DAPT (a γ-secretase inhibitor), which was found to significantly attenuate the release of HMGB1 in response to LPS. These findings indicate that Notch signaling activation favors LPS-induced HMGB1 release^124–126^.

### MAPK

MAPKs are signal transduction systems in eukaryotic cells that mediate the response of extracellular signals to intracellular signals. MAPKs are mainly composed of three subfamilies of highly conserved serine/threonine protein kinases, namely, extracellular regulated protein kinase (ERK), Jun N-terminal kinase (JNK) and MAPK14 (best known as p38). They conduct extracellular signals through a tertiary kinase cascade, specifically, extracellular signal → MAPK kinase (MKKK) → MAPK kinase (MKK) → MAPK^127^. MAPK family members divergently contribute to HMGB1 release in different inflammatory and injury models^128–131^. For example, the expression and release of HMGB1 is mediated through the p38 MAPK signaling pathway induced by rhesus rotavirus, while inhibition of p38 prevents HMGB1 release in cholangiocytes^131^. MAPK-mediated HMGB1 secretion is also implicated in endothelial inflammation, thus providing a potential therapeutic strategy for vascular diseases^132^. In vascular endothelial cells under high homocysteine conditions, the secretion of HMGB1 is positively regulated by the neuropilin 1 (NRP1)-MAPK14-MAPK pathway. Consequently, the knockdown of NRP1 by siRNA or administration of the NRP1 inhibitor ATWLPPR inhibits HMGB1 secretion in vivo and in vitro^132^. Together, these findings establish a functional role of MAPK in mediating HMGB1 secretion, although the detailed signal transduction mechanism remains unclear.

### STAT

STATs are a family of transcription factors (containing STAT1-6) that were initially identified by gene expression analysis triggered by IFN^133^. The STAT family plays a central role in signal transduction derived from different extracellular stimuli. STAT protein is usually located in the cytoplasm in an inactive form and is activated by tyrosine phosphorylation mediated by JAK1 and JAK2^134^. Phosphorylated STATs form homodimers or heterodimers, leading them to translocate to the nucleus and ultimately regulate the transcription of many target genes^135^. A large number of studies have shown that JAK-regulated activation of STAT1 and STAT3 plays a positive role in the expression, modification, and/or release of HMGB1 in different inflammation and injury models^136–139^. Disruption of the JAK-STAT signaling pathway inhibits HMGB1 release, thereby playing a protective role in sepsis and ischemia/reperfusion injury models^139,140^. Reciprocally, extracellular HMGB1 is able to trigger activation of the STAT1 and STAT3 pathways^139–143^. These findings reveal that an interplay exists between HMGB1 and STAT signaling in inflammation.

### TP53

HMGB1 and tumor protein P53 (TP53, best known as p53) can mutually regulate the signaling pathways they mediate and participate in many physiological and pathological processes^144,145^. For example, in cancer cells, HMGB1 and TP53 form a complex to regulate DNA repair and the balance between autophagy and apoptosis^146^. Knockout of TP53 increases the expression of cytosolic HMGB1 and induces autophagy, while depletion of HMGB1 in mouse embryonic fibroblasts promotes TP53 cytoplasmic localization and reduces autophagy^146,147^. Interestingly, after carcinogen administration, there is a cytoplasmic shift of HMGB1 in wild-type rat hepatocytes, and circulating HMGB1 levels are increased compared with levels in TP53^+/−^ rats, indicating that TP53 activation can control neoplastic inflammation by inducing HMGB1 release^148^. Moreover, HMGB1 also transports its nuclear binding target TP53 from the nucleus to the cytoplasm and participates in the resistance to sunitinib, indicating a direct interaction and cross-regulation between HMGB1 and TP53 in the presence of disease^149^.

### PPAR

Peroxisome proliferator-activated receptors (PPARs) belong to the nuclear hormone receptor family and contain three isotypes, namely, PPAR-α, PPAR-βδ, and PPAR-γ^150^. PPAR binds to specific ligands and activates the transcription of PPAR target genes, which plays a vital role in cell differentiation, tissue development, cell metabolism, inflammation, and tumor progression^151^. After administration of poly(I:C) or LPS, the secretion of HMGB1 in activated macrophages is negatively regulated by PPAR. Accordingly, treatment with rosiglitazone, a PPAR-γ ligand, decreases HMGB1 release and protects against sepsis in vivo and in vitro^152^. These findings provide new insights into the pleiotropic role of PPAR ligands in inhibiting lethal inflammation by targeting the release and activity of HMGB1^153^.

### Inflammasomes

Inflammasomes are polyprotein oligomers assembled in cells after the cells recognize DAMPs and pathogen-associated molecular patterns (PAMPs) and act as a platform for activation of canonical caspase-1 or noncanonical caspase-11 and the subsequent secretion of proinflammatory cytokines^154^. In addition to mediating maturation and release of the interleukin 1 (IL1) family, activated inflammasomes also promote HMGB1 release in immune cells^155–157^ or cancer cells^158^ through different signaling pathways. The phosphorylation and activation of eukaryotic translation initiation factor 2 alpha kinase 2 (EIF2AK2, also termed double-stranded RNA-dependent protein kinase [PKR]) is required for inflammasome-dependent interleukin 1 beta (IL1B) and HMGB1 release in macrophages^159^. In contrast, complement component 5a receptor 2 (C5aR2) deficiency restricts activation of the NLRP3 inflammasome and the release of HMGB1 in vitro and in vivo^160^. Moreover, M2 isoform of pyruvate kinase M2 (PKM2)-dependent glycolysis is involved in HMGB1 release through selective activation of NLR family pyrin domain containing 3 (NLRP3) and is absent in melanoma 2 (AIM2) inflammasomes in macrophages^161^. Therefore, pharmacological and genetic inhibition of inflammasome signaling pathways can attenuate the release of HMGB1 and protect mice from sepsis or ischemia/reperfusion damage^162–165^. Of note, NLRP3 can regulate HMGB1 release via an inflammasome-independent pathway^166^. Recent data demonstrated that in vitro HMGB1 release after inflammasome activation occurs after cellular rupture, which is likely inflammasome-independent in vivo^49^. Nevertheless, it is still difficult to distinguish between the active or passive release of HMGB1 mediated during inflammasome activation and subsequent pyroptotic cell death.

### Secretory lysosomes

In addition to mediating degradation, some lysosomes also act as secretory compartments, namely, secretory lysosomes. Secretory lysosomes are found in different types of immune cells and are Ca^2+^-regulated secretory organelles responsible for lysosome exocytosis^167^. It has been proposed that cytokine secretion can be regulated without leaders, such as IL1B and HMGB1, through lysosomal exocytosis^19^. Double immunofluorescence staining showed that HMGB1 colocalized with the lysosomal marker lysosomal-associated membrane protein 1 (LAMP1) but not the early endosomal marker early endosome antigen 1 (EEA1) in LPS-treated monocytes^168^. IFI30 lysosomal thiol reductase (IFI30, best known as gamma-interferon inducible lysosomal thiol reductase [GILT]) attenuates the formation of disulfide bonds in proteins, which assists in the complete unfolding of proteins for lysosomal degradation. NF-κB and STAT1, respectively, directly mediate the regulation of GILT expression induced by LPS and IFNG^169,170^. Depletion of IFI30 in immune cells (e.g., T cells and monocytes) can inhibit cytokine production (e.g., IL1B and TNF) and HMGB1 release in response to LPS, antigen exposure^169^, or mitochondrial oxidative damage^1,171,172^. Nuclear HMGB1 protein cytosolic translocation is then activated and associated with increased autophagy in IFI30^−/−^ fibroblasts^172^. These findings establish a role of IFI30 in regulating secretory lysosome-mediated HMGB1 release.

### Cell–cell interaction

The release of HMGB1 mediated by cell-cell interactions has been demonstrated in several studies. For example, the crosstalk between natural killer (NK) cells and dendritic cells (DCs) can cause HMGB1 release^173^. During vascular endothelial cell injury, platelets release a large amount of extracellular HMGB1 in the form of a disulfide isoform^174^, which plays a key role in neutrophil activation and thrombosis^174–176^. Clearance of apoptotic cells by phagocytic cells plays a role in the resolution of inflammation. Interestingly, the release of HMGB1 in macrophages induced by apoptotic cells has been considered one of the lethal mechanisms of apoptosis-mediated sepsis^177^. Impaired clearance of apoptotic cells also leads to the release of HMGB1, which in turn promotes TLR4-mediated cytokine production^178^. Thus, there is a dynamic relationship between the production and clearance of apoptotic cells in regulating HMGB1 release in macrophages. Moreover, the interaction between infiltrating uveitogenic T cells and retinal cells can induce rapid release of HMGB1 through the Fas cell surface death receptor (Fas)/Fas ligand inflammatory signaling pathway^179^. These findings further indicate that HMGB1 is a signaling mediator that coordinates the communication between different cells during an immune response.

### Other mechanisms

Targeted temperature management inhibits the extracellular release of HMGB1 in myocardial ischemic/reperfusion injury, indicating a role of temperature-sensitive mechanisms in HMGB1 secretion^180^. In addition, α7 nicotinic acetylcholine receptors (α7nAChRs) and α7nAChR-dependent cholinergic signaling are implicated in suppressing the release of HMGB1 and reducing the inflammatory response in acute lung injury^181^, highlighting the idea that HMGB1 is a mediator and target of neuroimmunity during critical illness. Interestingly, as a commonly used anti-inflammatory drug, corticosterone induces HMGB1 release via pannexin 1-dependent mechanisms in primary cultured rat cortical astrocytes^182^. The use of a large number of genetically defective mice (e.g., activating transcription factor 3 [ATF3]^−/−^^183^, C-C motif chemokine receptor 7 [CCR7]^−/−^^184^, IL17A^−/−^^185^, secretory leukocyte peptidase inhibitor [SLPI]^−/−^^186^, complement C3 [C3]^−/−^^187^, and ADAM metallopeptidase with thrombospondin type 1 motif 13 [ADAMTS13]^−/−^ mice^188^) in infection models further confirms the complexity and diversity of the HMGB1 release mechanism.

## Passive release of HMGB1

**Fig. 3:**
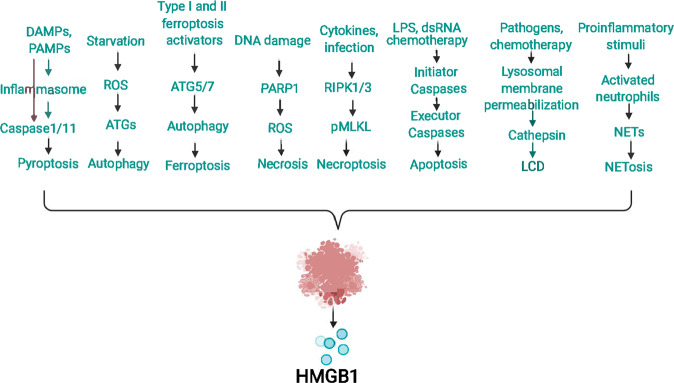
Passive release of HMGB1 during cell death. HMGB1 is a typical damage-associated molecular pattern (DAMP) that can be passively released during various types of cell death, such as pyroptosis, necrosis, apoptosis, necroptosis, ferroptosis, NETosis, and lysosome-mediated and autophagy-dependent cell death.

Cell death is generally divided into accidental cell death and regulated cell death. HMGB1 can be passively released after various types of cell death (such as necrosis, necroptosis, apoptosis, NETosis, lysosome-mediated cell death, pyroptosis, autophagy-dependent cell death, and ferroptosis) in response to various stimuli or damage. In this section, we discuss some key regulators of HMGB1 passive release (Fig. 3).

### PARP1

The PARP family comprises many enzymes responsible for catalyzing ADP-ribose transfer from nicotinamide adenine dinucleotide (NAD)^+^ to proteins during several types of stress^189^. PARP1 is the most abundant and characteristic PARP member in mammalian cell nuclei^190,191^. Extensive DNA damage has been identified as the most potent promoter of PARP overactivation, resulting in an extravagant demand for NAD^+^, ATP depletion, and subsequent necrosis^192^. Several groups of proteins (such as histones and HMG proteins) and certain transcription factors (such as TP53 and NF-κB) are substrates of PARP^193,194^. PARP1 activation is responsible for the active secretion of HMGB1 in response to LPS-challenged macrophages and mouse embryonic fibroblasts^37^. In addition, DNA-damaging drugs can induce HMGB1 release during necrosis through PARP1 activation. This PARP1-dependent translocation and release of HMGB1 has been observed in inflammation, fibrosis, and cell death^193,195^. In contrast, PARP inhibitors or genetic depletion of PARP1 in mouse embryo fibroblasts significantly attenuate alkylating DNA injury-induced HMGB1 translocation and release. Loss of HMGB1 in the cell will lead to excessive activation of PARP, thereby providing a positive feedback mechanism that promotes tissue damage.

### RIPK3

It has been well demonstrated that HMGB1 can be passively released by necrotic cells and causes sterile inflammation^2^. Previously, it was thought that necrosis was not a type of programmed cell death. However, recent progress has shown that necrosis that occurs in response to cytokines (such as TNF or type 1 or type 2 IFN), viral infection or chemotherapy may lead to highly programmed cell death, called necroptosis^196–198^. Receptor-interacting serine-threonine kinase (RIPK) family members (RIPK1 and RIPK3) and mixed-lineage kinase domain-like pseudokinase (MLKL) are key promoters of necroptosis due to their roles in the formation of necrosomes^2,199^. As a transition between TNF-induced apoptosis and necrosis, RIPK3 regulates necroptosis more specifically than RIPK1. RIPK3 knockout mice showed a reduction in sepsis and donor kidney inflammatory damage, which was found to be related to a decrease in DAMP release (including HMGB1)^200^. Pretreatment with the RIPK3 inhibitor necrostatin-1 can also reduce HMGB1 levels and protect animals from jejunal morphological injury^201^. In addition, RIPK3-regulated necroptosis is involved in dsRNA/poly (I:C)-induced HMGB1 release in a mouse model, which is the cause of inflammation during retinal degeneration^202^. In RIPK3^−/−^ mice, HMGB1 release was attenuated, and subsequent necrosis and inflammation were prevented, providing substantial protection against poly(I:C)-induced retinal degeneration^200^.

### Cathepsin

The concept of lysosome-dependent cell death (LCD) was first proposed by Christian de Duve, who famously identified cellular lysosomes as “recycle bins”^203^. This form of cell death is mainly mediated by lysosomal cathepsin; depends on leaked contents and the cell environment; and shows mixed characteristics of necrosis, apoptosis, and ferroptosis^204^. In response to a variety of stimuli, such as pathogen invasion and chemotherapy, the permeability of the lysosomal membrane increases, leading to cytoplasmic release of cathepsins and other hydrolytic enzymes^204^. Several members of the cathepsin family are involved in the release of HMGB1. For example, cathepsin B participates in L. pneumophila-induced LCD and HMGB1 release^205^. Cathepsin B-dependent NLRP3 inflammasome activation also contributes to nicotine-induced HMGB1 release and endothelial barrier dysfunction^206^. When cells are pretreated with specific cathepsin B inhibitors, cathepsin B may interact with nuclear proteins (such as core histone H3^207^ and HMGB1), thereby inhibiting cell death and subsequent HMGB1 release. In addition, transport of cathepsin B from the lysosome to the nucleus can cause DNA damage and subsequent release of HMGB1 for ferroptosis induction. Similarly, cathepsin D is involved in necroptosis-mediated HMGB1 release in immune cells^7^. In turn, HMGB1 can regulate cathepsin release and subsequent cell death^208^. Altogether, these studies indicate that cathepsin plays a significant role in regulating the release of HMGB1 during various types of cell death.

### Antioxidant enzymes

ROS are a double-edged sword in cellular processes, and their role changes depending on the threshold of oxidative stress. Excessive ROS accumulation can trigger cell damage and death^209^. In addition to inducing the active secretion of HMGB1, ROS can also promote the passive release of HMGB1 during various cell death modalities (such as necrosis, apoptosis, necroptosis, and ferroptosis)^210^. Accordingly, a group of antioxidant enzymes regulate the release of HMGB1 during ROS-related cell death. For example, dysfunction in superoxide dismutase 1 (SOD1) and superoxide dismutase 2 (SOD2) is implicated in promoting HMGB1 release during apoptosis^211–215^. Other antioxidant enzymes, such as peroxiredoxins^216^, glutathione reductase^172^, and thioredoxin^217^, also inhibit HMGB1 passive release in a context-dependent manner. Therefore, targeting HMGB1 with antioxidant compounds may be an attractive treatment strategy for tissue damage-related diseases.

### DNase

During cell death, DNA is degraded into fragments by different deoxyribonucleases (DNases), which participate in autoimmune diseases and other diseases. In apoptotic cells, DNA is degraded into nucleosomal units by DNA endonuclease (DNase-γ), while in necrotic cells, DNA is randomly degraded by extracellular DNase I or II^218^. NETosis is a form of regulated cell death first identified in neutrophils. In response to proinflammatory stimuli, neutrophils release their DNA and DNA-binding proteins (including HMGB1 and histones), which form neutrophil extracellular traps (NETs)^219^. The degradation of NETs caused by DNase significantly increases the release of HMGB1 during neutrophil activation^220^. NET-mediated activation of TLR4 and TLR9 signals can further enhance the release of HMGB1^221,222^. In addition to NETosis, DNase is also involved in regulating the release of HMGB1 in necrosis and apoptosis^223,224^. DNase inhibitors, such as DR396, can limit HMGB1 release during cell death^223,224^. Interestingly, bacterial-derived DNase can interfere with DNA stability through HMGB1, thereby impairing the type 1 IFN response in infection^225^. It is expected that the DNA sensor pathway may link cell death, HMGB1 release, and the immune response.

### Caspase

Caspases are a family of intracellular cysteine-aspartic proteases and are mainly involved in apoptosis and pyroptosis^226^. Two types of caspases, namely, initiators and executors, are involved in the apoptosis process. In response to stress signals, the initiator caspase is activated and then cleaves the executor caspase, which mediates apoptosis by hydrolyzing target proteins^227^. For example, caspase-8 is involved in cytokine processing and death receptor-mediated apoptosis signaling pathways^228^. PPAR-α activator fenofibrate-induced rat myocardial I/R damage is related to caspase-9-dependent mitochondrial apoptosis^229^. Caspase-9 can prevent accessibility of cytochrome c to complex III in the mitochondria, resulting in increased ROS production^230^. Moreover, apoptosis signals cause mitochondria to release cytochrome c and activate APAF1 (apoptosome), which then cleaves the proenzyme of caspase-9 into an active dimer form to mediate caspase-3 activation. Caspase-3 and caspase-7 mediate cleavage of the mitochondrial complex 1 protein p75, which promotes subsequent HMGB1 release during apoptosis. Whether other caspase cleavage substrates are related to the release of HMGB1 in apoptosis remains unknown. In addition, pretreatment with caspase inhibitors can significantly reduce the release of HMGB1 in experimental sepsis^177^. Unlike apoptosis, pyroptosis is typically triggered by activation of caspase-1 or caspase-11^231^. HMGB1 release is decreased by inhibiting caspase-1 activity during pyroptosis^165^. Compared with wild-type mice, the serum HMGB1 level in caspase-1/caspase-11 double-knockout mice is significantly reduced during lethal endotoxemia^156^. In addition, amino acids 67, 158, and 169 of HMGB1 can be cleaved directly by caspase-1 but not other caspases (-2, -3, -5, -7, -9, or -11)^232^. The caspase-1-modified A box (especially residues 23–50) is different from full-length HMGB1 in activity, and its binding to receptor AGER can remedy apoptosis-induced immune tolerance in sepsis^232^. All these results indicate that the processing and release of HMGB1 are regulated by caspases during inflammation and the immune response.

### ATG

Autophagy is a conserved degradation process that can remove unused proteins or damaged organelles by forming specific membrane structures (phagophores, autophagosomes, and autolysosomes). This process is mainly driven by a series of autophagy-related (ATG) proteins and usually plays a role in promoting cell survival^233^. Recently, HMGB1 secretion was reported to be regulated by heat shock protein 90 alpha family class A member 1 (HSP90AA1), Golgi reassembly stacking protein 2 (GORASP2)-mediated autophagy-based secretion machinery and the formation of multivesicular bodies^234^. However, excessive activation of the autophagic molecular machinery may cause cell death^233^. Iron-dependent ferroptosis is recognized as a form of autophagy-dependent cell death by degrading various anti-ferroptosis regulators^210^. In addition, autophagy-dependent secretion affects a number of extracellular factors, ranging from granule contents to inflammatory mediators. Knockdown of crucial autophagy proteins, such as ATG5, ATG7, and ATG12, significantly decreases extracellular HMGB1 release during necrosis^233^ or ferroptosis^235^. In addition, the secretion of HMGB1 by macrophages and fibroblasts also depends on ATG5 in response to LPS and starvation, which requires the participation of ROS signals^236^. Interestingly, HMGB1 release was observed in autophagy-deficient (ATG7^−/−^) hepatocytes and was found to be dependent on NFE2L2-regulated inflammasome activation. It is also worth noting that intracellular or extracellular HMGB1 is a promoter of autophagy through different mechanisms (e.g., by activating the phosphatidylinositol-4,5-bisphosphate 3-kinase catalytic subunit alpha [PI3K] complex or by inducing HSP27 expression), which may exacerbate autophagy-dependent ferroptosis^237–243^. The interaction between autophagy and HMGB1 provides an example of the complexity of the stress response during cell death.

### pH

Alkaliptosis is a pH-dependent cell death in cancer cells^244^. The drug JTC801 can induce alkaliptosis by activating the NF-κB pathway and inhibiting the expression of carbonic anhydrase 9 (CA9), a transmembrane metalloenzyme responsible for maintenance of intracellular pH^245,246^. Alkaliptosis is associated with the translocation and release of HMGB1, which is inhibited by the DNA repair pathway mediated by FA complementation group D2 (FANCD2)^247^. Once released by alkaliptotic cells, extracellular HMGB1 binds to the AGER receptor in macrophages, and then activates the stimulator of interferon response CGAMP interactor 1 (STING1, also known as TMEM173) pathway to produce pro-inflammatory cytokines (such as TNF and IL6)^247^. Thus, HMGB1 is an immune mediator of DNA sensor pathway activation induced by abnormal pH-mediated cell death.

## Conclusions and perspectives

HMGB1 is a position-dependent multifunctional protein. Inside the cell, nuclear HMGB1 regulates the structure and function of chromosomes, while cytoplasmic HMGB1 sustains autophagy. Outside the cell, HMGB1 is a mediator of inflammation, immunity, and the metabolic response. The release of HMGB1 is related to active or passive processes and can be adjusted at various levels. In particular, ROS and redox signals play a central role in driving the secretion and release of HMGB1 by coupling various posttranslational modifications. Targeting the release and activity of HMGB1 provides a strategy for the treatment of various diseases, especially infection and tissue damage. The following questions are worthy of our continued pursuit: How can we distinguish between the active secretion and passive release mechanisms of HMGB1? What is the main mechanism of HMGB1 secretion? Is there any difference in the activity of HMGB1 released by immune cells and nonimmune cells? What is the structural basis of HMGB1 lysosomal localization? Are the immune consequences of strategies that interfere with the translocation and release of HMGB1 different? How can we evaluate the acute and chronic effects of HMGB1 release on cell death-related immune responses? Compared with other DAMPs, what immune uniqueness does HMGB1 have? How can we develop an HMGB1-dependent combination drug strategy for disease treatment?
